# Alcances y limitaciones de la teleconsulta en pandemia de covid-19: relatos de profesionales de la salud del primer nivel de atención de la Ciudad Autónoma de Buenos Aires

**DOI:** 10.18294/sc.2024.4579

**Published:** 2024-02-16

**Authors:** María de las Nieves Ganiele, Mariela Alejandra Weisbrot, Andrea Melissa Sian, Julieta Milagros Carosella Reboredo, María Victoria Weisbrot, María Florencia Grande Ratti

**Affiliations:** 1 Médica. Investigadora, docente, Hospital Italiano de Buenos Aires, Ciudad Autónoma de Buenos Aires, Argentina. maria.ganiele@hospitalitaliano.org.ar Instituto Universitario Hospital Italiano de Buenos Aires Instituto Universitario Hospital Italiano de Buenos Aires Ciudad Autónoma de Buenos Aires Argentina maria.ganiele@hospitalitaliano.org.ar; 2 Magíster en Epidemiología, Gestión y Políticas de Salud. Investigadora, docente, Hospital Italiano de Buenos Aires, Ciudad Autónoma de Buenos Aires, Argentina. mariela.weisbrot@hospitalitaliano.org.ar Instituto Universitario Hospital Italiano de Buenos Aires Instituto Universitario Hospital Italiano de Buenos Aires Ciudad Autónoma de Buenos Aires Argentina mariela.weisbrot@hospitalitaliano.org.ar; 3 Estudiante de Medicina, Instituto Universitario Hospital Italiano de Buenos Aires, Ciudad Autónoma de Buenos Aires, Argentina. andrea.sian@hospitalitaliano.org.ar Instituto Universitario Hospital Italiano de Buenos Aires Instituto Universitario Hospital Italiano de Buenos Aires Ciudad Autónoma de Buenos Aires Argentina andrea.sian@hospitalitaliano.org.ar; 4 Estudiante de Medicina, Instituto Universitario Hospital Italiano de Buenos Aires, Ciudad Autónoma de Buenos Aires, Argentina. julieta.carosella@hospitalitaliano.org.ar Instituto Universitario Hospital Italiano de Buenos Aires Instituto Universitario Hospital Italiano de Buenos Aires Ciudad Autónoma de Buenos Aires Argentina julieta.carosella@hospitalitaliano.org.ar; 5 Socióloga. Investigadora, project manager, Instituto Universitario Hospital Italiano de Buenos Aires, Ciudad Autónoma de Buenos Aires, Argentina. victoriaweisbrot@gmail.com Instituto Universitario Hospital Italiano de Buenos Aires Instituto Universitario Hospital Italiano de Buenos Aires Ciudad Autónoma de Buenos Aires Argentina victoriaweisbrot@gmail.com; 6 Doctora en Ciencias de la Salud. Investigadora, Consejo Nacional de Investigaciones Científicas y Técnicas. Docente, Instituto Universitario Hospital Italiano de Buenos Aires, Ciudad Autónoma de Buenos Aires, Argentina. maria.grande@hospitalitaliano.org.ar Instituto Universitario Hospital Italiano de Buenos Aires Instituto Universitario Hospital Italiano de Buenos Aires Ciudad Autónoma de Buenos Aires Argentina maria.grande@hospitalitaliano.org.ar; Consejo Nacional de Investigaciones Científicas y Técnicas

**Keywords:** Consulta Remota, Telemedicina, Atención Primaria de Salud, Argentina, Remote Consultation, Telemedicine, Primary Health Care, Argentina

## Abstract

El objetivo fue explorar alcances y limitaciones de la teleconsulta en pandemia, desde la perspectiva de médicos y médicas del primer nivel de atención del Hospital Italiano de Buenos Aires, una institución privada ubicada en la Ciudad Autónoma de Buenos Aires. Se realizó un estudio cualitativo con diez entrevistas semiestructuradas individuales entre enero y abril de 2022. Los tres grandes tópicos emergentes fueron la transición a la virtualidad, la accesibilidad y el nuevo modelo de atención. Los obstáculos se relacionaron con la implementación masiva, forzada y no planificada de las teleconsultas. Los principales beneficios fueron brindar atención durante el aislamiento-distanciamiento y evacuar dudas epidemiológicas. Se destacan cambios en estrategias de atención, encuadre de las consultas, intercambio entre colegas, criterios de derivación y de pedido de estudios complementarios, y en los perfiles de consultantes. Surgió un sobreuso del sistema por parte de las personas, y una banalización del momento de la consulta. El auge de las tecnologías de la comunicación e información indudablemente permitió dar continuidad a los procesos asistenciales en salud, pero no reemplaza la presencialidad y se requieren lineamientos normativos para su continuidad.

## Abstract (en)

The aim was to explore the scope and limitations of teleconsultation during the pandemic from the perspective of primary care physicians at the Hospital Italiano de Buenos Aires, a private institution located in the Autonomous City of Buenos Aires. A qualitative study was conducted with ten individual semi-structured interviews between January and April 2022. The three major emerging topics were the transition to virtuality, accessibility, and the new care model. Obstacles were related to the massive, forced, and unplanned implementation of teleconsultations. The main benefits included providing care during isolation-distancing and addressing epidemiological doubts. Changes were highlighted in care strategies, consultation frameworks, exchange among colleagues, referral criteria, requests for complementary studies, and in the profiles of those seeking consultations. A misuse of the system by individuals and a trivialization of the consultation moment emerged. The rise of communication and information technologies undoubtedly allowed the continuity of healthcare processes, but it does not replace in-person care, and normative guidelines are needed for its continuity.

## INTRODUCCIÓN

La *telesalud*, definida como la prestación de servicios a través de teléfono o video, se ha utilizado en la atención médica durante décadas^1^. Originalmente, se desarrolló como respuesta al envejecimiento poblacional y la escasez de camas hospitalarias, con la intención de producir una atención más rentable y mejorar las posibilidades de tratamiento de las personas^2^. Fue ideada para aproximar los servicios de salud a las poblaciones residentes en lugares remotos y/o con escaso acceso a los recursos de salud^3^. Posteriormente, la American Telemedicine Association apoyó su expansión y uso de aplicaciones de telesalud, monitoreo remoto, y manejo de enfermedades en el hogar^4^.

Recientemente, la Organización Mundial de la Salud reconoció que la *salud digital* debe formar parte de las prioridades, para lograr beneficiar a las personas de una manera ética, segura, fiable, equitativa y sostenible^5^. Adicionalmente, planteó que debería desarrollarse, respetando los principios de transparencia, accesibilidad, escalabilidad, replicabilidad, interoperabilidad, privacidad, seguridad y confidencialidad^5^. Para la Agenda 2030 de Naciones Unidas, se establecieron 17 Objetivos de Desarrollo Sostenible, dentro de los cuales se remarcó la expansión de las *tecnologías de la información y la comunicación* (TIC) y la interconexión mundial, debido a que facilitan: acelerar el progreso humano, superar la brecha digital, y desarrollar las sociedades del conocimiento^6^. Según el Plan Nacional de Telesalud 2018-2024 del ex Ministerio de Salud de Argentina, la *telemedicina* se define como la provisión de servicios de salud por parte de los profesionales sanitarios, donde la distancia es un factor crítico, y se utilizan las TIC. 

Por otro lado, las pandemias plantean importantes desafíos para la prestación de servicios de salud, especialmente en países vulnerables, como los de América Latina^7^. Por ende, el covid-19 aceleró su adopción en muchos entornos de atención a nivel mundial^8^. Como consecuencia, modificó los modos de relacionarnos, la habitualidad del rostro visible, los modos de trabajo, y el cuidado y la atención de la salud. Asimismo, permitió un auge de las TIC, garantizando la continuidad de los procesos asistenciales en salud pese a las medidas de aislamiento y distanciamiento social frente a la situación epidemiológica, ya que la principal vía de transmisión era a través de las secreciones respiratorias por contacto social^9^. 

### Medidas epidemiológicas adoptadas en Argentina

Luego de reportarse los primeros casos de infección por covid-19, las respuestas iniciales involucraron el aislamiento de pacientes positivos y la evaluación de los viajeros que arribaban del exterior^10^. Posteriormente, fue declarado estado de emergencia sanitaria nacional, momento en el que todos los países de la región implementaron una serie de medidas para hacer frente a la pandemia, relacionadas con la disminución del contacto social, y la implementación del teletrabajo y de la teleeducación^10^. Estas medidas fueron variando de acuerdo a factores como la gravedad de la propagación del virus, la capacidad de recursos sanitarios, la infraestructura existente y las políticas gubernamentales. Cabe señalar que las estrategias evolucionaron a lo largo del tiempo a medida que se obtenía más información sobre el virus, su propagación y la eficacia de las diferentes alternativas.

**Figura 1:**
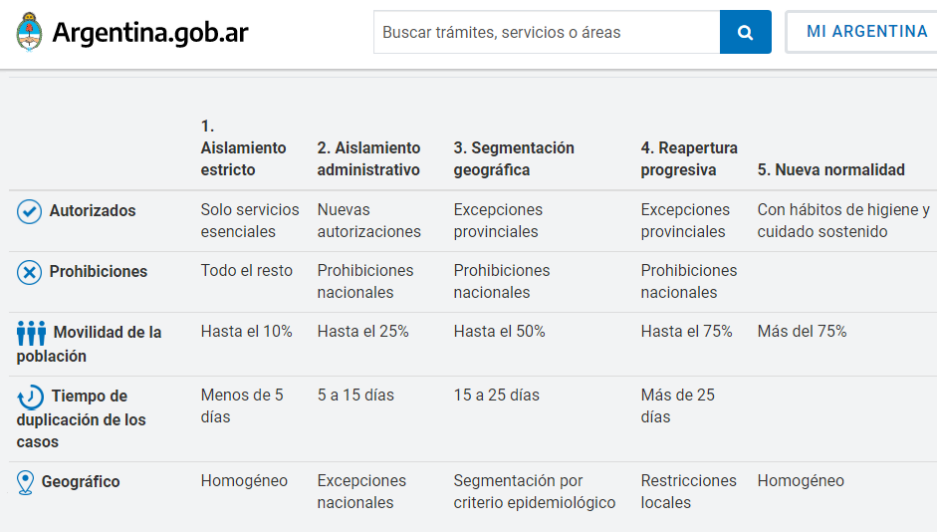
Medidas sanitarias por la pandemia de COVID-19 en Argentina: Cinco fases de aislamiento estricto, aislamiento administrativo, segmentación geográfica, reapertura progresiva y nueva normalidad. 2020-2021.

El gobierno argentino decretó el Aislamiento Social Preventivo y Obligatorio (ASPO), que obligaba a todas las personas a permanecer en sus hogares, salvo aquellas personas que trabajaran en actividades definidas por el mismo decreto como “esenciales”. Solo podían salir para el abastecimiento de alimentos y medicamentos, o en casos de extrema necesidad (y habiendo previamente tramitado un permiso de circulación). El ASPO abarcó inicialmente todo el territorio nacional desde el 20 de marzo hasta el 26 de abril 2020 inclusive. Luego, desde el 27 de abril 2020 en adelante se establecieron medidas graduadas en cinco fases (Figura 1) y segmentadas territorialmente (aislamiento o distanciamiento), según lo exigiera la situación sanitaria de cada lugar. Las fases 1 a 3 fueron fases de cuarentena (aislamiento), mientras que las fases 4 y 5 fueron fases de distanciamiento (sin cuarentena). Dependiendo la situación en cada región, provincia, ciudad e incluso barrio, la fase podría variar en el sentido de una mayor o menor movilidad, incluso retrotrayendo a una fase anterior, en caso de que empeorara la situación.

Fuente: Portal oficial del Estado argentino.

### Implementación de teleconsultas en Argentina

La *teleconsulta sincrónica*, entendida como el encuentro en tiempo real entre médico y paciente por videoconferencia, surgió como el principal mecanismo para responder a las demandas no urgentes de salud^11^. Resultó una herramienta valiosa a la hora de promover el distanciamiento social y evitar la sobrecarga del sistema sanitario durante la pandemia, principalmente para permitir la continuidad de las consultas ambulatorias programadas (o no) y sortear las dificultades para acceder al sistema de salud, especialmente en el ámbito público^12^. Hubo varias experiencias nacionales publicadas, se desarrolló un consultorio de teledermatología del Hospital de Agudos José María Ramos Mejía^12^, se instauró seguimiento ambulatorio de pacientes covid-19 en Misiones^13^ y en un hospital privado ubicado en La Matanza^14^, se implementaron teleconsultas para embarazadas y madres en Córdoba^15^, se diseñó e implementó un chatbot de atención médica^16^, y se reportaron teleconsultas cardiológicas en el Hospital El Cruce Néstor Kirchner^17^^,^^18^, y de pacientes con enfermedad vascular crónica en el Hospital Italiano^19^.

### Marco de teleconsultas en el Hospital Italiano de Buenos Aires

La institución cuenta con un sistema de información de salud desarrollado internamente, con más de 20 años de experiencia^20^. Desde la era prepandemia ya contaba con un *Portal Personal de Salud* integrado a la historia clínica electrónica, cuyas funcionalidades incluían: programación de citas (agenda de turnos presenciales), visualización de resultados de estudios/pruebas, sistema de mensajería médico-paciente segura que facilita el intercambio de información de salud asincrónico (denominado *radiomensajes*), gestión de medicamentos (recetas electrónicas), y algunas teleconsultas. Las experiencias previas de teleconsultas fueron limitadas y restringidas a pocas especialidades médicas, como aquellas de dermatología que requerían un servicio de almacenamiento y envío de información relacionada con la salud por parte del paciente al médico especialista^21^; o las citas de primera vez con especialistas en oncología ortopédica^22^. Sin embargo, fueron principalmente de modalidad asincrónica, y/o para la atención no programada (por ejemplo: programas de telegripe y teletriaje)^23^^,^^24^.

Durante el ASPO, esta nueva modalidad de atención creció masivamente, reemplazando incluso el 100% de las visitas presenciales durante un tiempo, debido a que se suspendieron transitoriamente todas las agendas programadas^25^, y para entonces la mayoría de los profesionales de la salud y de los pacientes desconocían esta herramienta. En consecuencia, la pandemia estimuló la utilización en forma casi imperativa de las teleconsultas sincrónicas, pudiendo entonces garantizar la continuidad de cuidados para la atención ambulatoria y programada^19^^,^^26^. Se migraron 14.000 turnos presenciales a virtuales en menos de 3 días^25^. Las teleconsultas fueron encuentros sincrónicos entre médico y paciente mediante una videoconferencia, diseñada internamente (con video, audio y chat), cuya funcionalidad fue integrada a la historia clínica electrónica (para el profesional) y al portal personal de salud (para el paciente).

**Figura 2:**
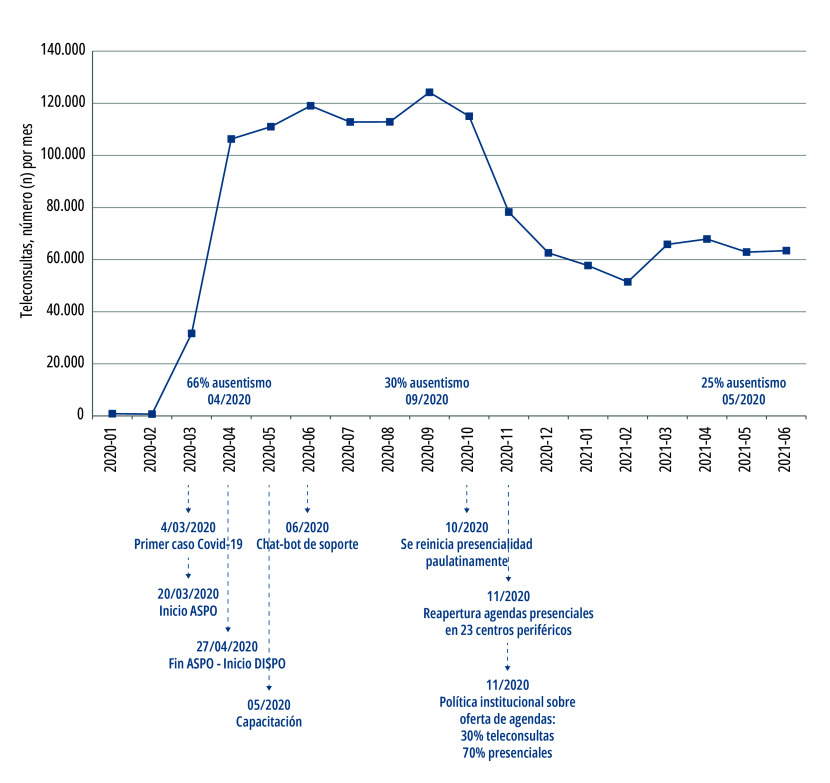
Número de turnos de teleconsultas programadas por mes, durante 2020 y 2021, y tasa de ausentismo (expresada en porcentaje). Hospital Italiano de Buenos Aires, Ciudad Autónoma de Buenos Aires, 2022.

Este sistema se basó en la premisa de no necesitar la instalación de ningún software, y con acceso seguro a través de una *virtual private network* (VPN) o red privada virtual (se habilitaron 1.215 VPN a personal de salud), lo que permitió ofrecer este servicio sin comprometer la seguridad de la red hospitalaria^25^. Existió inicialmente un gran salto en el número de teleconsultas, llegando a realizarse más de 4.000 diarias (Figura 2), que impulsaron un programa de telemedicina cuyo avance hasta el momento había sido lento y escalonado^25^.

Fuente: Elaboración propia. Nota: Debajo el eje X se muestran los hitos importantes a nivel institucional, que dan cuenta del marco temporal. ASPO= Aislamiento Social, Preventivo y Obligatorio. DISPO= Distanciamiento, social preventivo y obligatorio.

### Modelos de atención en atención primaria de la salud

En el año 2001, Barbara Starfield^27^ definió las funciones de la atención primaria, entendida como el primer nivel de atención, como: el primer contacto, la continuidad, la coordinación y la integralidad. El *primer contacto* supone la idea de que cada vez que surge un problema nuevo de salud se acude a un centro o profesional concreto, siendo la *accesibilidad* el elemento estructural necesario. La *continuidad* observa la relación personal a largo plazo entre el usuario y el profesional o centro de salud, que debe facilitar el desarrollo de una relación basada en la confianza y en el conocimiento de la persona y su familia (relación médico-paciente). La *coordinación* es la función de enlace entre los servicios de atención. Por último, la *integralidad* se refiere a la característica de ofrecer y articular los servicios (por ejemplo, sean orgánicos, funcionales o sociales) que la población necesita. Estos conceptos fueron retomados luego por varios autores^28^^,^^29^^,^^30^.

Por otro lado, la *medicina centrada en el paciente* es un modelo de atención que describe la relación médico-paciente como un vínculo dinámico entre ambos, donde se explora tanto la enfermedad como la dolencia, que permite comprender a la persona en forma *integral* y en su *contexto* a través de la búsqueda del diálogo y el entendimiento^31^^,^^32^. De esta manera, la práctica médica como intervención científica y moderna no puede prescindir del trabajo reflexivo e individualizado, centrado en la persona^33^.

Si bien las tecnologías digitales están transformando el sector de la salud en todo el mundo, varios aspectos de este campo emergente de la ciencia aún deben comprenderse adecuadamente. El presente trabajo, se propuso explorar los alcances y las limitaciones que experimentaron las teleconsultas desde la visión de médicos y médicas del primer nivel de atención, desde el contexto de excepción por covid-19 hasta la actualidad.

## METODOLOGÍA

Se realizó un estudio cualitativo exploratorio-descriptivo, realizado en el Hospital Italiano de Buenos Aires (HIBA), un centro de salud de alta complejidad, ubicado en la Ciudad Autónoma de Buenos Aires, Argentina. En esta entidad privada se atienden personas con diferentes coberturas, prepagas u obras sociales, así como también cuenta con una prepaga propia de la institución. Los afiliados a esta última cuentan con un *médico de cabecera* asignado, que funciona como médico de primer nivel de atención, dado que es la puerta de acceso al sistema, sostiene un modelo longitudinal de atención y coordina los cuidados del paciente. 

En cuanto a los criterios de selección de las y los profesionales del presente estudio se consideró que sean médicos y médicas de cabecera del *primer nivel de atención* del HIBA, que hayan atendido teleconsultas programadas durante la pandemia, como especialistas en medicina interna, clínica, medicina familiar, medicina general y/o pediatría.

Se realizó un muestreo intencional de diez profesionales, mediante técnica de bola de nieve, hasta obtener la saturación del discurso^34^. El rango etario de los mismos fue de 31 a 67 años, siete eran de sexo femenino y llevaban entre 2 a 42 años de ejercicio de la profesión. Con respecto a la especialidad médica, seis pertenecían al servicio de Medicina Familiar y Comunitaria; dos a Clínica Médica, y dos a Pediatría. Mientras que solo cuatro habían utilizado teleconsultas prepandemia (para consultas de guardia no programadas, vigentes desde 2019 en la institución), todos utilizaron esta herramienta durante el aislamiento-distanciamiento social, y nueve continuaban prestando atención con esa modalidad al momento de la entrevista. Con respecto a los honorarios, la mayoría cobraba por prestación, por lo que era variable de acuerdo al número de pacientes atendidos efectivamente, y solo una persona tenía contratación en relación de dependencia (por hora trabajada).

El trabajo de campo se llevó a cabo entre enero y abril de 2022. Se utilizó la técnica de entrevistas semiestructuradas con guía de preguntas *ad-hoc*. Los interrogantes que guiaron el estudio giraron en torno a conocer cómo afectó la modalidad de consulta virtual a su práctica cotidiana (relación con las personas atendidas, estilo de atención, juicio clínico, capacidad diagnóstica) y qué estrategias generaron para dar respuesta y continuar con la atención de las personas en el contexto de la pandemia covid-19.

Las entrevistas fueron individuales, virtuales y duraron entre 45 y 60 minutos. El material fue audio-grabado y luego transcripto. El análisis de la información se basó en la codificación del contenido (mediante códigos predefinidos y abiertos) y la generación de conceptos mediante la inducción analítica. Primero se codificó línea por línea, y cada documento fue evaluado por doble revisión. Luego, se analizó de manera transversal en un proceso estructurado. Los pasos para el análisis, según Taylor-Bogdan^35^, se basa en un trabajo iterativo, avanzando y retrocediendo al revisar tres pasos: descubrimiento, codificación y relativización en paralelo, donde el objetivo es desarrollar una comprensión profunda. Las categorías emergentes pueden representar nuevos temas, conexiones inesperadas o patrones significativos que no se habían identificado en el marco teórico o en las preguntas de investigación iniciales, pero permiten una comprensión más completa y rica del fenómeno estudiado (Tabla 1).

**Tabla 1:** Principales categorías analíticas, predeterminadas (*) y emergentes (^), sobre los alcances y las limitaciones de las teleconsultas desde la visión de médicos y médicas del primer nivel de atención. Hospital Italiano de Buenos Aires, Ciudad Autónoma de Buenos Aires, 2022.

Categoría	Descripción	Subcategorías
Transición a la virtualidad (*)	Cambio o desplazamiento de los servicios médicos tradicionales ofrecidos en consultas presenciales hacia plataformas y tecnologías digitales para brindar atención médica a distancia.	(*) Requerimientos (dispositivos, Internet, ambiente de trabajo)
		(*) Entrenamiento / experiencia / curva de aprendizaje
		(*) Infraestructura institucional
		(^) Soporte técnico
		(^) Funcionalidades (certificado o receta digital)
		(^) Normas institucionales
		(^) Financiamiento y convenios de pago
Accesibilidad (*)	Facilidad y disponibilidad con la que los individuos pueden acceder a los servicios de salud básicos, preventivos y de atención inicial que ofrecen los sistemas de salud. Más allá de disponibilidad geográfica (presencial) y horaria (virtual), incluyen otros factores como la disponibilidad de recursos, la asequibilidad, la aceptabilidad cultural, la información comprensible y la capacidad de recibir atención sin discriminación.	(*) Descentralización hospitalaria
		(*) Analfabetismo digital
		(*) Adultos mayores
		(^) Pacientes del interior
		(^) Sobreutilización
		(^) Ausentismo
		(^) Límites desdibujados
		(^) Sobrecarga para médicos y médicas
Nuevo modelo de atención (*)	Se refiere a un enfoque actualizado y adaptado de prestación de servicios médicos a través de la tecnología para la atención primaria de la salud.	(*) Perfiles de pacientes
		(*) Motivos de consulta
		(*) Capacidad diagnóstica y nuevas estrategias para suplir el examen físico
		(*) Encuadre del nuevo tipo de vinculación médico paciente
		(^) Continuidad del cuidado, seguimiento y monitoreo
		(^) Paciente oportunista
		(^) Vínculos menos cercanos y más fríos
		(^) Trabajo solitario, pérdida de contacto con colegas
		(^) Pérdida del marco habitual para la entrevista médica (privacidad)

Fuente: Elaboración propia a partir de Taylor-Bogdan^35^.

### Consideraciones éticas

Todas las personas participantes convocadas para las entrevistas dieron su consentimiento informado oral y su decisión voluntaria al inicio de cada entrevista. Para garantizar la confidencialidad se utilizaron etiquetas durante el almacenamiento seguro, y el plan de trabajo contó con la aprobación correspondiente por el comité de ética institucional (CEPI#5708).

## RESULTADOS

### Transición de presencialidad a virtualidad

Debido al ASPO dictaminado en marzo del 2020, el HIBA migró la atención programada de pacientes de la presencialidad a la virtualidad, a gran escala. La teleconsulta funcionó como un medio para garantizar el acceso a la continuidad del cuidado médico sin incumplir las restricciones vigentes. Estas teleconsultas tuvieron por objeto reemplazar el encuentro entre médico-paciente que se daba en el marco del consultorio presencial.

Los médicos y las médicas de cabecera del estudio vivieron las primeras semanas con gran ansiedad. Al migrar la agenda de turnos a la forma virtual, debieron desarrollar habilidades para adaptarse a esta novedosa estrategia de atención dado que no contaban con entrenamiento previo.

*Me pareció algo nuevo, que también yo me tenía que adaptar, además de todo el contexto* [...] *era como una forma de atención nueva.* (E1, clínica médica)

*Nunca trabajé en mi casa, no tengo esa rutina, mis herramientas* [...] *por ahí ya no era el estetoscopio o el tensiómetro, pero mi computadora tenía que funcionar, tener cargadores, tener mousse, tener wifi. Tuve que instalar telefonía e Internet, y un plan ilimitado para el celular.* (E9, medicina familiar)

Esta nueva modalidad de atención y su abrupta implementación, trajo consigo varias dificultades. Las y los profesionales entrevistados debieron generar una infraestructura acorde. Fue necesario adaptar el espacio personal para generar un ambiente propicio de trabajo, donde no sufrieran interrupciones, contaran con señal de Internet y dispositivos tecnológicos adecuados (esto se complicaba especialmente en las viviendas donde habitaban niños pequeños). También debieron invertir en tecnología, en redes de Internet o en infraestructura mobiliaria.

*Fue complicado la verdad, la recuerdo como una situación estresante, algo nuevo que no conocíamos, con bastantes obstáculos y dificultades* [...] *no tenía computadora para mí porque no me alcanzaba con los chicos acá online, me tuve que comprar una.* (E3, pediatr*ía*)

*Yo desde el día que dijeron que nos íbamos a quedar un poco más en casa me metí a comprar una silla más cómoda y me armé el consultorio en dónde tengo el escritorio.* (E5, medicina familiar)

*Yo tengo hijos chiquitos, así que siempre estaba el tema de los ruidos* [...] *y que bueno intentar organizar en familia de que ese rato era yo estoy trabajando y no puedo estar además con mi familia que no siempre es sencillo.* (E4, clínica *médica*)

Por otro lado, algunos componentes de las consultas -como hacer recetas, actualizar la evolución del paciente en la historia clínica, expedir certificados médicos- que suelen darse todos juntos en lo presencial, en lo virtual se abordan como componentes separados. Normalmente, en el tiempo de una consulta presencial se suelen resolver la mayor parte de las tareas; sin embargo, en la consulta virtual, quedan como cuestiones pendientes que se van resolviendo a lo largo del día generando mayor demanda de tiempo de trabajo en los médicos. Poco a poco el sistema institucional fue incorporando nuevas funcionalidades, como recetas electrónicas y certificados virtuales, lo que agilizó las consultas virtuales equiparándolas, en este aspecto, a las presenciales.

*Tenes que ver cómo tener recetarios en papel, en algunas cuestiones como recetas es bastante incómodo, sacar la foto, mandarla por mail, que cómo te salió... si los pacientes tienen un recetario en particular no te lo pueden traer, y ellos igual tienen que ir en forma presencial.* (E1, clínica médica)

*Creo que al inicio de la pandemia las recetas me sobrecargaban mucho más porque ahora el portal te lo permite hacer más fácil, en realidad hay convenios con muchas prepagas y el sistema funciona.* (E1, clínica médica)

*Todo esto se empezó a aceptar virtual: recetarios virtuales, certificados virtuales, eso me parece que es una ventaja enorme.* (E9, medicina familiar)

Médicas y médicos entrevistados relatan que fueron testigos de la transición y adaptación que vivieron las personas. Las primeras semanas del consultorio virtual, a pesar de que las agendas estuvieran plenas de turnos asignados, las personas no estaban enteradas de que el turno médico seguía garantizado en dicha modalidad. Una vez que esta información circuló, desconocían la forma de acceder.

Para resolver este desencuentro, la estrategia que utilizaron las y los profesionales en las primeras semanas fue llamar por teléfono a cada paciente que contaba con un turno y que no se había conectado a la consulta.

*Hasta que los pacientes se enteraban cómo tenían que usar el sistema hubo un mes que no hice nada* [...] *lo que me pasaba era que tenía la agenda llena, pero nadie entraba, y era porque les habían pasado el turno de presenciales a virtuales, pero la gente ni se enteraba.* (E2, medicina familiar)

*Al principio, te acordás que teníamos que llamar nosotros a los pacientes para avisarles. Entonces había un montón de consultas que ya se las resolvías por teléfono. La gente mayor no entendía nada, y había mucho miedo.* (E8, medicina familiar)

Con el correr de los días algunos pacientes conseguían conectarse al turno virtual. Sin embargo, era frecuente que las consultas no pudieran desarrollarse dentro de la plataforma oficial, dadas las numerosas dificultades en mantener la comunicación efectiva: problemas de conexión a Internet, problemas con la infraestructura institucional (plataforma, historia clínica, portal de salud) y falta de dispositivos personales adecuados. Los profesionales reaccionaron a este inconveniente utilizando canales alternativos de comunicación, como el teléfono, WhatsApp, Meet o Zoom. En un momento intermedio, el sistema de telemedicina fue muy útil para saber qué paciente estaba en lista y presente para iniciar la consulta, pero luego la comunicación se completaba por otros canales.

*Al principio había mucha dificultad con la comunicación, la verdad que terminaba llamando al paciente por teléfono o a veces hacia un meet o un zoom.* (E3, pediatr*í*a)

*Al inicio dependió del médico, o sea de la voluntad de uno, de las cosas, de las herramientas, de la computadora, de internet.* (E4, clínica *médica*)

De a poco las personas usuarias fueron entendiendo el sistema, la plataforma de teleconsulta se fue perfeccionando y las consultas pudieron desarrollarse casi en su totalidad por este medio.

*Me acuerdo que festejé una vez que pude hacer toda una teleconsulta entera, no me acuerdo en qué momento pero a los 20 días o al mes fue mejorando.* (E3, pediatr*í*a)

### Accesibilidad

Los médicos y las médicas de cabecera de nuestro estudio refieren que la teleconsulta, a su vez, facilitó el acceso a la atención médica sin necesidad de trasladarse, como las personas del interior o de otros países. Una vez flexibilizadas las medidas epidemiológicas (entre octubre y noviembre de 2020 se reabrieron las agendas presenciales en el hospital central y se habilitaron los 23 centros ambulatorios periféricos), varios usuarios continuaron eligiendo esta modalidad a distancia. En ese contexto, la política institucional fue mantener un máximo de 30% de horas semanales de atención bajo modalidad virtual, pudiendo mantener las agendas con ambas alternativas. Esta descentralización permitió ahorrar en gastos de transporte y tiempo de traslado, que resultó relevante tanto para profesionales como para pacientes.

*A mucha gente le puede ahorrar mucho tiempo de transporte no sólo a personas que viven en el interior también a la gente que vive en Capital o en Gran Buenos Aires.* [...] *también es cómodo a veces no ir al hospital y estar en mi casa.* (E1, clínica médica)

En tanto la accesibilidad tecnológica, la implementación masiva de la tecnología interoperable para establecer comunicaciones mediante videoconferencias (software o hardware) tuvo una rápida evolución en la institución. En tan solo pocos días, se virtualizaron todas las agendas presenciales. La persona se conectaba desde su portal personal de salud y el médico o la médica de cabecera a través de la historia clínica electrónica, utilizando cualquier dispositivo (computadora, celular o tablet). Esta comunicación se estableció con una videollamada institucional, comunicación simultánea bidireccional de audio y vídeo y la existencia de un chat.

*Podían hacer consultas desde su casa, y nosotros con la plataforma del hospital, teníamos la teleconsulta a los 20 días de iniciar la pandemia* [...] *para abril 2020 migraron todas las agendas a la virtualidad.* (E3, pediatr*í*a)

*Obviamente el sistema no funcionaba bien porque estaba saturado. Con el diario del lunes la contingencia del sistema estuvo a la altura de lo que se podía hacer en ese momento, porque de repente todos en el hospital empezamos a hacer eso por una misma plataforma en simultáneo.* (E5, médico de familia)

Todas las personas entrevistadas experimentaron problemas técnicos de diferente índole (por ejemplo: para permitir el acceso de los micrófonos y cámaras al sistema operativo, uso de micrófonos y webcam no integrados, ausencia de pruebas técnicas), pero con el tiempo se notó una mejora progresiva. La institución prestó acompañamiento, soporte técnico, y brindó tutoriales.

*Primero odiaba el sistema de teleconsulta porque no funcionaba, porque andaba mal y ahora digo ¡Qué hermoso que es esto! Eso sí cambió claramente.* (E5, medicina familiar)

*Fue mejorando durante la pandemia el tema de la información, de cómo debería la gente ingresar al portal, hay tutoriales. Está muy bien ejemplificado. El hospital se tomó todo el trabajo de que las personas tengan menos dificultad y se fueron facilitando cosas.* (E6, medicina familiar)

En tanto el acceso según cobertura en salud, la teleconsulta fue una posibilidad habilitada inmediatamente para los afiliados al Plan de Salud del Hospital Italiano. Las obras sociales y otras prepagas no gozaron de convenios con el hospital para brindar atención sincrónica a distancia, lo que dejó a sus afiliados por fuera del sistema oficial de las teleconsultas. Las médicas y los médicos de cabecera del estudio respondieron a esta situación continuando con la atención de estos pacientes por fuera de las vías institucionales (correo electrónico, WhatsApp, teléfono) y sin percibir remuneración económica por su trabajo.

*La incertidumbre del principio... esto (teleconsulta a usuarios de obras sociales y otras prepagas) no me lo va a pagar nadie nunca jamás en la vida.* (E4, clínica *médica*)

La Gerencia Comercial y el Departamento de Atención Ambulatoria del hospital tardó dos meses en establecer convenios administrativos, logrando así el reconocimiento de esta modalidad por parte de obras sociales y prepagas para el cobro. Sin embargo, cada profesional fue adoptando tanto canales formales como informales de comunicación de acuerdo a sus posibilidades y conveniencias. Indudablemente, se tomaron medidas que afectaban a la cobertura sanitaria, el sistema de financiación, la remuneración de los proveedores sanitarios, el acceso de los pacientes a las pruebas y a la atención^36^.

A la vez, esta situación de incertidumbre se perpetuó debido al rechazo (o falta de aceptación) por parte de las obras sociales y prepagas de las recetas electrónicas. La dificultad radicaba en una teleconsulta exitosa y efectiva, pero completamente ineficiente para el paciente cuyo motivo de consulta era la necesidad de una receta en papel para la medicación crónica, por ejemplo. La Ley 27553 sobre recetas electrónicas o digitales estableció que la prescripción y dispensación de medicamentos, y toda otra prescripción, puedan ser redactadas y firmadas a través de firmas manuscritas, electrónicas o digitales, en recetas electrónicas o digitales, en todo el territorio nacional, y puedan utilizarse plataformas de teleasistencia en salud. Esta situación de excepcionalidad estuvo vigente por la pandemia desde julio de 2020^37^, como alternativa que garantizara el derecho de acceso a los tratamientos médicos con la imposibilidad de poder concurrir a las consultas para recibir las recetas, por el aislamiento obligatorio dispuesto en el contexto de la emergencia sanitaria por la pandemia de covid-19, a través del Decreto de Necesidad y Urgencia 297 de 2020^38^. Sin embargo, la receta foto o papel escaneada fue un instrumento temporario, debiendo regresar indefectiblemente a la receta en papel con la firma manuscrita o receta electrónica con firma digital (a validar en farmacia) desde el 1 de marzo de 2023, a través de la Resolución 304/2023^39^. 

El acceso a la teleconsulta fue especialmente dificultoso para los adultos mayores. En esta población el nivel de desconocimiento de las nuevas tecnologías (analfabetismo digital) generó una barrera y, tanto médicos como pacientes, debieron desarrollar estrategias para lograr la comunicación efectiva. Al principio las médicas y los médicos entrevistados llamaban sistemáticamente a todos las personas mayores que tenían turnos. La familia de estos pacientes funcionó de intermediaria para lograr la comunicación. Con el tiempo, varios adultos mayores fueron aprendiendo a utilizarla por sus propios medios.

*Sentí mucha más presencia de los familiares, hijos, nietos que ayudaban a los abuelos en las conexiones.* (E8, medicina familiar)

Luego de un tiempo (para mayo de 2021) varias personas mayores volvieron a elegir la presencialidad como modo de comunicación con sus médicos, temporalidad que coincide con la vacunación completa prioritaria para ellos. Cabe recordar que la vacunación se inició el 29 de diciembre de 2020 luego de recibir un suministro de 300.000 dosis, que fueron destinadas al personal de salud^40^. Un total de 29.985 personas adultas recibieron al menos la primera dosis entre enero y mayo 2021, de los cuales el 85% eran adultos mayores^40^. Sin embargo, según las médicas y los médicos del estudio, algunas personas mayores tuvieron gran resistencia a salir de la casa (por el miedo al contagio), y continuaron utilizando la teleconsulta.

*Hay personas mayores que no les gusta ni siquiera la devolución por teléfono pudiendo hacerlo necesitan venir a verme, porque para muchos la entrevista médica es la oportunidad de un motivo social, también una excusa ocupacional.* (E7, medicina familiar)

*Tengo un pequeño núcleo que han salido 2-3 veces en toda la pandemia, a vacunarse y que no quieren salir y resultaron fans de la teleconsulta.* (E5, medicina familiar)

La teleconsulta, al no estar sujeta al espacio físico determinado por el hospital, permitió que las y los profesionales pudieran realizar ajustes de sus horarios de consultorio según su conveniencia.

*Muchas veces está el problema del lugar físico y que uno no llega a atender muchísimos pacientes en poco tiempo... no ten*é*s los consultorios suficientes, entonces la teleconsulta puede, digamos, salvar ese problema.* (E1, clínica médica)

*El horario que tenía de presencial de adolescencia no me servía ya hace un montón porque era muy tarde, y la única forma de adelantarlo era tener teleconsultas.* (E10, pediatr*í*a)

*Por último, facilitó la realización de entrevistas familiares, por ser más simple que varios participantes coordinen horarios y se conecten en simultáneo. Tuve una teleconsulta con la madre, que no la conocía salvo por la teleconsulta* [...]. *Tuve una entrevista familiar con la madre y el hermano* [...] *en forma virtual.* (E2, medicina familiar)

### Nuevo modelo de atención

La telemedicina además de imprimir cambios sobre la accesibilidad, también creó nuevas formas de relacionarse entre médicos y pacientes.

#### Perfiles de pacientes

En contraposición con la presencialidad, los entrevistados reconocieron mayor frecuencia en las consultas de hombres, adolescentes, adultos jóvenes y personas que viven en otras provincias/países; y disminuyeron las consultas de las personas mayores. En el ámbito pediátrico refirieron que la mayoría de las teleconsultas eran con la mamá (no papá), y sin el niño presente en la videollamada.

*Por ahí de repente aparecen más hombres que no suelen ser los que más consultan al médico, pero lo tenían más accesible.* (E2, medicina familiar)

*Creció la cápita, por ahí más pacientes jóvenes que se manejan bien con lo que es Internet y el sistema y lo vieron como una oportunidad.* (E4, clínica *médica*)

En el ámbito del primer nivel de atención, la relación con las personas en la consulta es una herramienta de trabajo fundamental y una estrategia de atención. El bagaje de conocimientos sobre la historia longitudinal y contextual, y la relación construida en la presencialidad, fue reconocido por las médicas y los médicos entrevistados como un plus a la hora de la teleconsulta. En cambio, las relaciones nacidas en la virtualidad se caracterizaron por sentirse menos cercanas, más frías. La individualidad de la persona queda menos registrada en la memoria de las y los médicos entrevistados, lo que debilitó la generación de la relación médico-paciente. Luego del ASPO fue una estrategia frecuente citar presencialmente a las personas cuyas primeras consultas fueron virtuales, para generar esa cercanía. Algunos comparan el turno presencial con “conocer al paciente”, es decir que previo a ese encuentro no había una relación real.

Por otro lado, distinguieron un nuevo perfil de “paciente oportunista”, entendido o caracterizado como aquella persona que aprovecha ciertas oportunidades en el ámbito de su atención sanitaria, específicamente por hacer sobreuso de las teleconsultas, por sacar turnos sobre la hora, con cualquier médico, temas de consultas más banales que no hubieran sido motivos suficientes para asistir a un turno presencial.

*La teleconsulta para mi ofrece dos grupos de pacientes: el paciente que yo atiendo de toda la vida y como el modelo de atención de medicina familiar valora mucho hablar de lo contextual, eso no lo pierdo y lo sigo usando exactamente igual. Pero apareció un grupo de pacientes nuevos que es el oportunista, que encuentra un turno libre, nunca lo viste en tu vida. Con ese paciente soy como una persona que hace demanda espontánea.* (E7, medicina familiar)

*Es una vinculación mucho más fría. O sea, soy técnicamente correcto y respondo al cuestionamiento, resuelvo lo que quiere que le resuelva y demás, pero los pacientes nuevos que adquir*í*s virtualmente, los registrás menos.* (E2, medicina familiar)

*Hubo muchas más consultas efímeras de personas con las que no logré establecer vínculo.* (E9, medicina familiar)

#### Motivos de consulta

En la etapa del ASPO (desde 20 de marzo hasta el 26 de abril 2020 inclusive), los médicos entrevistados relataron que las consultas eran “rapiditas”, por motivos puntuales, mayormente dudas o necesidades informativas ligadas a la pandemia. Fueron recurrentes las consultas sobre la vacuna antigripal y la vacuna antineumocócica, la utilidad del uso del tapabocas y otras medidas de cuidado^41^. La relación entre la vacuna antigripal, la vacuna antineumocócica y la pandemia por covid-19 se basa en la importancia de la prevención y el fortalecimiento del sistema inmunológico para reducir la carga sobre los sistemas de salud. Por ejemplo, disminuye la incidencia de la gripe, ayuda a prevenir la confusión entre la gripe y el covid-19 (síntomas similares), y reduce el riesgo de complicaciones respiratorias que podrían agravar la situación de una persona con neumonía bacteriana que también contraiga covid-19.

Se reconoce que cada etapa de la pandemia tuvo su motivo de consulta preponderante. Consultas por eclosión de información y luego eclosión de casos (“*¿soy contacto estrecho?, ¿me hisopo?*”). A la vez, se abordaron por teleconsulta temas de educación, relacionados con conductas saludables o de cuidado. Por ejemplo, surgieron consultas por preocupaciones sobre desinfección de las verduras, circulación de personas externas al hogar (como cuidadoras y empleadas domésticas), prevención en la infancia, entre otras.

*Al principio era “te quiero pedir tal y tal cosa”, como que era una duración mínima, como te digo rapidito rapidito. Se me hacía como cuando no había teléfono celular y hablabas a larga distancia, como una sensación de que estaba como limitado, y ahora hay gente que le encantó la teleconsulta.* (E5, medicina familiar)

*Y estaba focalizada en el virus y a las medidas de cuidado: como usar el alcohol, como lavar la verdura, como limpiar el piso, ¿qué hago con la empleada?* (E2, medicina familiar)

*En algún momento también llegó a ser monotema el “estuve en contacto estrecho con tal con tal con tal o tengo síntomas, ¿me hisopo?* (E5, medicina familiar)

La mayoría de los médicos resaltaron haber tenido gran número de consultas por síntomas relacionados a la salud mental (mayormente síntomas depresivos y de ansiedad). Durante las medidas de aislamiento y gran parte de la etapa de distanciamiento social, muchas personas necesitaron hablar de su angustia, incertidumbre, soledad y utilizaron esta herramienta como una situación social de charla con alguien que se interesara por ellos.

*Mucha crisis vital, mucha depresión reactiva, mucha cosa anímica.* (E6, medicina familiar)

*Sirve quizás para las consultas de seguimiento de la esfera emocional, que hubo muchas durante la pandemia, me eran más fáciles hacerlas virtual. Si la persona tenía un espacio de intimidad y podía hablar tranquilamente, seguimiento de medicación de psicofármacos o de cuadros de depresión, ansiedad los podía seguir virtualmente.* (E9, medicina familiar)

Las médicas y los médicos entrevistados refirieron que la teleconsulta tiene gran utilidad para pedidos de estudios complementarios y devolución de los resultados, realización de recetas y seguimiento de problemas psicosociales. Uno de ellos refirió que prefiere devolver resultados de estudios de manera virtual para que lo presencial quede disponible para “el que se siente mal”.

*Me parece que es muy cómoda cuando tenés que resolver cosas concretas y ver resultados de estudios. O pacientes que te piden, quiero hacerme un chequeo y para eso piden la teleconsulta, me parece que es una consulta súper práctica, cómoda, y que ahorra tiempo para todos.* (E8, medicina familiar)

Históricamente, los dos principales motivos de consulta en pediatría eran control de salud y patologías agudas infectocontagiosas típicas de la infancia. Durante la pandemia disminuyó sustancialmente el número de consultas relacionadas con estas últimas, al no compartir espacios presenciales con otros niños. Los temas que reconocieron poder abordar mediante teleconsulta fueron los que no requerían examen físico, como control de esfínteres, límites, alimentación y angustia en los niños, anticoncepción en adolescentes, dudas sobre vacunación contra el covid-19.

*Consultas que como para hablar de límites, control de esfínteres, preocupación por la alimentación o algo concreto que les estaba pasando de angustia, lo veo bueno porque no se tenían que trasladar al hospital y lo podían hacer consultas desde su casa.* (E3, pediatr*í*a)

Parte de las médicas y los médicos entrevistados remarcaron que a través de la teleconsulta (tanto en la etapa de aislamiento como de distanciamiento social) tuvieron la posibilidad de sostener la atención longitudinal de sus pacientes, acompañándolos en situaciones de mucha incertidumbre y soledad. Cuanto mayor y más antigua era la relación médico-paciente establecida, se destacó más esfuerzo o dedicación por parte de las y los profesionales para acompañarlos. La teleconsulta sincrónica transmitía una sensación de mayor cercanía que un mensaje a través del portal o correo electrónico.

En la actualidad, en las modalidades mixtas de atención, se aprecia la telemedicina como un habilitador de seguimiento cercano y cómodo para problemas que requieren consultas más frecuentes.

*Valoro el poder comunicarse visualmente con una persona, fue como un momento de salvataje para ellos. Poder llevar el mensaje de tranquilidad, cuando uno además puede ser observado por el otro, para la gente que confía en uno era muy importante y era más aliviador que el llamado telefónico o responder la mensajería.* (E7, medicina familiar)

*Me gusta que la atención sea mixta, porque puedo ofrecer un espacio de seguimiento más cercano, para muchos es muy útil, o les gusta controlarse principalmente por este método.* (E1, clínica médica)

#### Capacidad diagnóstica y nuevas estrategias

De las entrevistas se destaca que la capacidad de realizar un diagnóstico se vio facilitada por la relación médico-paciente consolidada. En consonancia con Schraiber^33^, las interacciones regulares a lo largo del tiempo hacen que el médico tenga un conocimiento más profundo del historial, los síntomas y las peculiaridades individuales de la persona, lo que indudablemente facilita la identificación y comprensión de los problemas de salud. Schraiber reitera este concepto donde menciona “el arte del médico se expresa en las decisiones que el profesional toma frente a las exigencias del caso [...] El arte es, en ese sentido, fruto de la experiencia personal”^33^. En contraposición con este concepto, los “nuevos pacientes” (conocidos solo por teleconsulta) podrían presentar desafíos adicionales en el proceso diagnóstico.

*Con los pacientes conocidos, uno ya conoce hasta su modo de enfermar, su modo de sanar, y uno tiene un mapa mental del paciente y sus procesos, entonces con esos pacientes era mucho más fácil hacer teleconsulta.* (E5, medicina familiar)

Todos concuerdan que la falta de examen físico limitaba la evaluación de varios problemas de salud como respiratorios, gastrointestinales, progresión de peso en niños, entre otros. Esta limitación impulsó nuevas estrategias para suplirlo, como la realización de un interrogatorio más exhaustivo y la participación activa de las propias personas en el proceso de atención (compartir fotos o enfocar la cámara para mostrar lesiones en piel, controlar sus signos vitales -saturación de oxígeno, temperatura, presión arterial-, filmar comportamientos de los hijos que les resultaban extraños, registrar el peso de los bebés por el método de peso diferencial).

*En la pandemia destinaba más tiempo a hacer un interrogatorio más profundo, para tener más tranquilidad desde la teleconsulta, insistir un poquito más con los síntomas, que lo relate mejor.* (E1, clínica médica)

*Al principio el lenguaje fue la única herramienta que tenía, porque como no tenía examen físico tenía que hacer casi todo verbal con mucha colaboración (del paciente).* (E9, medicina familiar)

*Tuve que adquirir un montón de estrategias nuevas,* [...] *la más fácil es “sáquese una foto y póngala en el portal así veo su manchita”.* [...] *a veces tuve que explicar maniobras de elongar el cuello a través de la cámara.* (E7, medicina familiar)

*Decía “filmalo y lo veo”* [...] *¿Esto es un tic o una mioclonía o un movimiento normal? ¿me tengo que preocupar? con algún recurso visual, un video.* (E3, pediatr*í*a)

Otra estrategia que se obtuvo del corpus fue la habilitación de espacios dentro del hospital para completar la evaluación en casos puntuales. De esta forma, los servicios médicos habilitaron un “consultorio de contingencia” para hacer frente a la derivación por colegas que requerían complementar la teleconsulta con un examen físico. En cuanto a las y los pacientes pediátricos, se aprovechó el momento de la vacunación para llevar a cabo el examen físico del control del niño sano y en las personas adultas se pospusieron controles de salud en población sana.

Una tercera estrategia fue hacer uso de la opinión de especialistas (interconsultas o derivaciones) o complementar el examen con estudios. El proceso de pensamiento diagnóstico a través de la teleconsulta no se vio afectado en quienes contaban con más años de experiencia profesional; mientras que aquellos con menos años de experiencia manifestaron sensación de inseguridad y recurrieron a derivaciones con especialistas y solicitud de estudios complementarios con mayor frecuencia que en la presencialidad, para descartar o afirmar diagnósticos.

*Considero que tengo la espalda suficiente (años de experiencia) como para poder manejar cualquier entrevista médica y decidir telefónicamente o por teleconsulta si es para ambulatorio, guardia, internación o terapia, me parece que con pocas palabras uno puede discriminar eso.* (E7, medicina familiar)

*Ante la imposibilidad de verlo, entonces por ahí hubo casos de pedidos extras (de estudio complementarios).* (E4, clínica *médica*)

La atención virtual desarticuló momentos informales de intercambio con colegas (consultas entre pares que surgen espontáneamente al compartir el mismo ambiente físico de trabajo) y disminuyó la posibilidad de resolver dudas. Como respuesta, se crearon redes de apoyo social que colaboraron a poner en común nuevas estrategias de atención y afianzar los criterios de manejo; lo que quitó peso y estrés a las decisiones individuales (grupos de WhatsApp y reuniones periódicas virtuales).

*En forma presencial hubiera podido consultar con otro pediatra en el consultorio de al lado como red de apoyo social.* (E10, pediatría)

#### Encuadre del nuevo tipo de vinculación médico-paciente

Un aspecto destacado que surge del corpus es la ruptura del modelo tradicional de práctica asistencial que generó la telemedicina al eliminar barreras geográficas y temporales. Con respecto a esto, Schraiber menciona que:

…aunque la relación entre los profesionales y los pacientes sea de autoridad técnica, ¿cómo proponer disciplinas de vida, a no ser en consonancia con el otro, estableciendo una complicidad en torno de la autoridad técnica? Los propios profesionales expresan esta cuestión a través de la noción de “adhesión”. El paciente que adhiere es aquel que es colaborativo y, éticamente, comparte los mismos valores que el médico.^33^

Sin embargo, la mayor accesibilidad a turnos y comunicación asincrónica (mensajería del portal, correo electrónico, WhatsApp) se tradujo en una “banalización” del momento de la consulta, término conocido y utilizado por la bibliografía^42^^,^^43^. Esta idea sugiere que, debido a la naturaleza remota y a menudo rápida de las teleconsultas, algunas personas pueden no tomarlas tan en serio como una consulta médica tradicional en persona. Algunos ejemplos que dan cuenta de este fenómeno son: menor compromiso o seriedad (lugar inapropiado para teleconsulta como conduciendo un auto u oficina compartida sin confidencialidad, ausentismo frecuente sin cancelación), menor profundidad en la comunicación (en cuanto a calidad y cantidad de la información compartida, que puede parecer escasa), percepción de rapidez y superficialidad. Si bien ya existían con anterioridad canales institucionales e informales de comunicación asincrónica a distancia, se empezó a sentir un sobreuso^41^. Los profesionales manifestaron percibir que los pacientes sacaban turnos de manera frecuente, por temas de salud poco relevantes, y que el ausentismo era algo frecuente y usual. De esta forma, la teleconsulta profundiza el aspecto mercantil del modo en que los pacientes se relacionan con la medicina, donde la intervención médica es como cualquier otro consumo o prestación de servicio.

Lo peor son los ausentes. Y capaz que es la facilidad que tienen para sacar el turno desde la casa, y de no tener que ir al hospital. (E1, clínica médica)

Consideraron que la teleconsulta modificó de manera negativa el marco de la entrevista clínica. Relataron una disminución del respeto por el momento de la consulta (pacientes sin remera), en espacios inadecuados (manejando, caminando por la calle), presencia de familiares que alteraban la privacidad (interrumpiendo o escuchando sin haberse presentado).

*También me ha pasado de tener que pedirle a los pacientes que respeten este espacio porque me han llamado desde autos y decirles “¡No podes estar manejando porque esto es peligroso, estacioná!”* (E9, medicina familiar)

En la consulta presencial el momento de atención está delimitado por el espacio-tiempo y la finalización de la consulta queda claramente establecida. En cambio, en este nuevo contexto (de la generalización de la telemedicina) se dio una ilusión de que las médicas y los médicos estaban a disposición de las demandas de las personas usuarias en todo momento, casi como un *continuum*. El contenido amplio y el número de consultas asincrónicas varió causando un aumento de las horas de trabajo del profesional no remuneradas. Médicos y médicas tuvieron que dedicar tiempo extra para organizar un nuevo encuadre, “poner límites” a las personas, y establecer pautas para la comunicación. Diferenciar entre consultas que se pueden resolver de forma asincrónica de aquellas que requieren de un encuentro sincrónico (por teleconsulta); de los usos adecuados y oportunos del WhatsApp (en aquellos profesionales que compartían su celular) y de la mensajería a través del portal de salud. Sobre este punto hubo un consenso sobre la falta de lineamientos institucionales y el establecimiento de reglas hospitalarias con respecto al uso de las herramientas digitales.

*Hay pacientes que no saben usar la herramienta de mensajería, y de repente hay como “testamentos”, que escriben y se contestan y vuelvan a preguntar y se responden solos.* (E5, medicina familiar)

*Ahora usan la mensajería como si fuera el WhatsApp, entonces nos llueven mensajes todo el día.* (E7, medicina familiar)

*Me gustaría que hubiera una normativa. Cuando tengamos más experiencia, creo que vamos a ir construyéndolas y eso me haría sentir más cómoda.* (E9, medicina familiar)

## DISCUSIÓN

El contexto covid-19 impulsó una aceleración de procesos de transformación digital. En el ámbito de la salud, nos llevó a adoptar herramientas informáticas, sin capacitación tanto a profesionales como usuarios. Para los médicos del primer nivel de atención, esto implicó el cambio de la tradicional atención presencial a la utilización de teleconsultas. Alineados con Martínez *et al*.^44^, ha significado un reto para el sistema sanitario y para la atención primaria, donde se paralizó prácticamente toda la actividad programada (visitas, seguimientos de pacientes crónicos, etc.), y se mantuvo únicamente la atención urgente, desplazando la actividad mayoritaria de los profesionales a la atención virtual y telefónica. Tal como mencionan Giménez *et al*.^25^ este proceso representó un enorme desafío, ya sea por la dimensión social, emocional, personal, profesional, organizacional, y la propia complejidad del sistema de salud. Se generó así una nueva reorganización del espacio cotidiano de trabajo dando lugar a nuevas dinámicas de interacción entre el médico, el saber, la tecnología y el paciente^33^.

Los tópicos emergentes del análisis del corpus empírico permitieron la pesquisa de alcances y limitaciones de esta herramienta. Surgieron obstáculos relacionados con la implementación masiva, forzada y no planificada. Se detectaron factores relacionados con aspectos tecnológicos (conectividad, dispositivos o analfabetismo digital), aspectos estructurales (lugar, energía eléctrica, convivientes o interrupciones), institucionales (protocolos, recomendaciones, normativas o convenios con prestadores), clínicos (motivos de consulta inapropiados a la modalidad o restricciones en el examen físico) y vinculares (contacto más distante con pacientes conocidos solo por teleconsulta, trabajo solitario con menor intercambio con colegas, etc.). En forma consistente, Habib *et al*.^45^ reportaron que la mayor barrera percibida (68,7%) fueron las dificultades técnicas; mientras que Cunha *et al*.^46^ identificaron las siguientes: falta de acceso a servicios de Internet; falta de acceso a dispositivos tecnológicos; disparidades raciales, étnicas y etarias; baja alfabetización digital; falta de recursos económicos; barreras del idioma; falta de cobertura por parte del seguro médico; preocupaciones sobre la privacidad y confidencialidad de los datos; disparidades geográficas; y la necesidad de pruebas diagnósticas complementarias o la entrega de resultados de las pruebas. En nuestro estudio, la edad y la cobertura de seguro médico también fueron variables que estuvieron presentes de manera reiterada en los relatos. Si bien esto fue una solución para muchos, otros no tenían acceso a estas tecnologías o tuvieron problemas para usarlas, particularmente las personas mayores, subgrupo ya conocido y mencionado en la evidencia científica^47^. Otras limitantes mencionadas en la bibliografía fueron la ausencia del contacto presencial en detrimento de la relación médico-paciente, pérdida de la exploración física, la falta de acceso a Internet o dispositivos electrónicos digitales, y la carencia de personal capacitado con un enfoque en el uso de tecnologías de la información^48^. 

A pesar de esto, los principales beneficios fueron evitar desplazamientos y traslados innecesarios que permitieron cumplir con medidas epidemiológicas de aislamiento-distanciamiento, y responder a las necesidades informativas epidemiológicas de los pacientes (medidas preventivas y de cuidado, necesidad de testeo para covid-19, manejo de contacto estrecho y vacunación)^41^. A su vez, esta modalidad favoreció la accesibilidad para pacientes del interior o del exterior, y facilitó la continuidad del cuidado de las personas, por ejemplo, con patologías y medicación crónica. En consonancia con estos hallazgos, Loza *et al*.^47^, al explorar las experiencias de salud de las personas durante el encierro, concluyen que para contrarrestar la falta de accesibilidad al sistema de salud se utilizaron recetas digitales para medicamentos crónicos y soluciones tecnológicas para consultas médicas en línea de emergencia o no programadas. En nuestro estudio, un factor clave fue, sin duda, la infraestructura institucional preexistente (plataforma, historia clínica electrónica, portal de salud, el Servicio de Informática Médica), ya que la teleconsulta pudo implementarse en tiempo récord, cumpliendo los protocolos establecidos y con el resguardo de los derechos de los involucrados, a través de procesos seguros que garantizaron la privacidad y/o confidencialidad (adecuado tratamiento de información sensible, consentimiento, comunicación efectiva). Como mencionan Martínez *et al*.^44^, para potenciar la atención no presencial, es imprescindible disponer de la tecnología que garantice un buen funcionamiento de la visita por vía telemática (telefónica, correo electrónico, videollamada) de forma segura y confidencial y capaz de absorber la demanda surgida.

La masificación de las tecnologías de la información y la comunicación (TIC) y el mayor uso de teleconsultas desestructuraron el modelo de atención primaria clásico, que representa la base del sistema sanitario y se caracteriza por la accesibilidad, la longitudinalidad, la integralidad, la resolutividad, la coordinación y la continuidad asistencial^28^^,^^29^^,^^30^. No solo modificó la forma de abordar las consultas, sino que también cambió sustancialmente la forma de comunicación (inmediatez), y la lógica de contacto entre médico y paciente. Como describen Jiménez-Carrillo *et al*.^49^ puede conducir a un empeoramiento de la relación médico-paciente en términos de más quejas, un trato de pacientes más impersonal, por lo que no es una herramienta para todas las personas (ni pacientes ni médicos). También Schraiber reflexiona sobre cómo estás herramientas de comunicación pueden actuar como una barrera más en la relación médico-paciente, y convoca a encontrar los límites y el sentido para favorecer la interacción entre las personas^33^. 

Esta masificación permitió garantizar mayor flexibilidad en horarios de atención y/o más disponibilidad de turnos. Asimismo, aquellas consultas en donde la palabra se destaca, abordadas a través del diálogo e interrogatorio, la utilización de esta herramienta es una opción más viable. En este sentido, la teleconsulta evidenció tener gran utilidad para solicitud de estudios complementarios y devolución de dichos resultados, realización de recetas electrónicas, y seguimiento virtual de problemáticas psicosociales. En consistencia con estos hallazgos, Jiménez-Carrillo *et al*.^49^^)^ mencionan que la teleconsulta puede tener una capacidad resolutiva alta para ciertos motivos de consulta como son los trámites burocráticos, gestión de pruebas diagnósticas o de medicación.

Sin embargo, la facilidad de acceso a la teleconsulta podría generar un aumento del ausentismo, representando un costo sanitario significativo, en un contexto con demoras para obtener un nuevo turno^50^. Inicialmente, para abril de 2020, la tasa de ausentismo global (consultas presenciales y virtuales) fue del 66%, probablemente explicada por la falta de comunicación al paciente sobre la migración de agendas a teleconsultas y/o falta de entrenamiento en esta nueva modalidad. Sin embargo, desde agosto de 2020 se siguió manteniendo en 30% de manera persistente, lo que representa un importante obstáculo en la costo-efectividad de la asistencia sanitaria^51^.

Con el paso del tiempo (para octubre o noviembre de 2020), la teleconsulta permitió complementar la presencialidad, y actualmente (enero de 2024) se sigue ofreciendo una modalidad mixta (o híbrida) de atención, lo que permite potenciar lo mejor de cada una, y facilitar la libertad de elección individual a las personas. Los resultados del presente estudio alertan sobre la importancia de tener un contacto presencial para otorgar entidad individual a los pacientes. Aspectos del compartir el espacio físico, de apreciar la comunicación no verbal, de tener charlas cortas e informales sobre temas ajenos al mundo médico, influyen en la construcción de un vínculo más personalizado. Schraiber describe la medicina como “la práctica basada en interacciones y en la confianza recíproca entre médicos y pacientes y entre profesionales”, en la que los médicos viven la despersonalización del cuidado como un conflicto^33^. En la teleconsulta al ver un recorte de la imagen del paciente (menor corporeidad), la comunicación tiende a la despersonalización del cuidado generando una mayor tensión del trabajo médico. Esto se acentúa si la comunicación es telefónica, en la que directamente no hay imagen. 

Indudablemente, se logró un aprendizaje (por parte de médicos y de pacientes) en el uso de esta modalidad, pero sigue existiendo el desafío de asegurar una relación de la misma calidad y respeto que en una consulta presencial, tanto desde la personalización de la atención, como en relación con el abordaje clínico acorde a los conocimientos científicos actualizados y la evidencia disponible.

En forma consistente con la literatura, un estudio mexicano alertaba en 2017 sobre la lentitud de la conexión a Internet como principal barrera, y reportaba la gran ventaja del acceso a la atención^52^. Otro estudio aportaba las principales ventajas: mayor prestación de servicios, reducción de distancia y costos, adopción por parte de pacientes de un rol más activo^53^; y coincidieron con las barreras: infraestructura de las TIC, capacitación, brecha digital, y privacidad^53^.

El campo de la telesalud conlleva promesas históricas para mejorar el acceso, el costo y la calidad de la atención^54^. Sin embargo, no está claro hasta qué punto está cumpliendo esas promesas, ya que todavía está surgiendo la evidencia científica necesaria para justificar este éxito. El desarrollo de recomendaciones y estándares para las teleconsultas es un proceso importante y valioso para ayudar a garantizar la prestación segura y eficaz de atención médica de calidad^55^. La creación de pautas es un tema fundamental para los financiadores y los tomadores de decisión, que cada vez más las están adoptando e integrando en las regulaciones y políticas^55^.

Caben mencionar algunas limitaciones inherentes al diseño y metodología de este trabajo. En primer lugar, es unicéntrico (en un hospital privado de alta complejidad), lo cual limita la generalización de las observaciones a otros subsectores de salud (porque la prestación de salud se limita a personas con prepagas y obras sociales, dejando fuera relatos de experiencias del sistema público). En segundo lugar, los resultados se limitan a la visión de médicos y médicas del primer nivel de atención, por lo que estarían faltando otras voces y miradas necesarias e importantes (pacientes, tomadores de decisión). En tercer lugar, los resultados podrían estar sujetos a potenciales sesgos: de información (de recuerdo), o de selección (muestreo intencional). Pese a todo esto, creemos que el principal aporte radica en la generación de información para el nivel local, con gran validez interna. Otra fortaleza podría estar relacionada con la indagación de percepciones, preocupaciones y vivencias de las y los profesionales del primer nivel de atención en un contexto epidemiológico con un enfoque hospitalocentrista^56^. En ese contexto, sostuvieron la actividad asistencial de manera completamente invisible, llevando a cabo tareas esenciales para la comunidad y la población, como gestión del miedo y de la incertidumbre, acompañamiento de adultos mayores que vivían solos, gestión de la enfermedad de manejo ambulatorio, dando respuesta a las necesidades informativas de las personas. Adicionalmente, cabe destacar que el corpus empírico resultó más que suficiente para responder a los objetivos, pese el tamaño muestral reducido, no siendo necesarias reformulaciones de estrategias.

## CONCLUSIONES

La tradicional atención presencial en el primer nivel de atención siempre tuvo la función de estar presente en el cuidado más allá del encuentro en consultorio. Todas otras formas de comunicación históricas (mensaje en un beeper para recurrir a teléfono, radiomensaje por portal, llamado telefónico) ya eran telemedicina, pero tal vez no lo apreciábamos.

Sin embargo, se concluye que las teleconsultas abren un nuevo campo de conocimiento sobre el que es necesario seguir reflexionando. Por un lado, se requiere mejorar la difusión de guías de recomendaciones existentes. Por el otro, desarrollar protocolos de atención para el abordaje de los problemas de salud más frecuentes en atención primaria, mediante la teleconsulta. La enseñanza y el aprendizaje de esta herramienta podría ser brindado a través de cursos de formación continua para profesionales.

Quisiéramos alertar sobre la posibilidad de que esta herramienta genere mayor ausentismo, con el impacto que conlleva al sistema y a la salud de las y los pacientes; las áreas grises de un plexo normativo que respalda esta práctica (conformado por leyes y acuerdos locales), aún pendientes de ser abordadas; y la profundización de la despersonalización del cuidado en detrimento de la producción de servicios en un marco de vínculos de confianza.

Por último, será necesario fortalecer la investigación desde las visiones de las y los pacientes, otras especialidades médicas no contempladas por este trabajo, otros centros de atención (especialmente públicos, con diferentes perfiles socioeconómicos); o incluso un cambio de estrategia metodológica hacia lo cuantitativo permitiría producir y explorar datos complementarios, que colaboren al entendimiento y caracterización del contexto.
